# Explicit Solutions of a Gravity-Induced Film Flow along a Convectively Heated Vertical Wall

**DOI:** 10.1155/2013/475939

**Published:** 2013-12-25

**Authors:** Ammarah Raees, Hang Xu

**Affiliations:** State Key Lab of Ocean Engineering, School of Naval Architecture, Ocean and Civil Engineering, Shanghai Jiao Tong University, Shanghai 200240, China

## Abstract

The gravity-driven film flow has been analyzed along a vertical wall subjected to a convective boundary condition. The Boussinesq approximation is applied to simplify the buoyancy term, and similarity transformations are used on the mathematical model of the problem under consideration, to obtain a set of coupled ordinary differential equations. Then the reduced equations are solved explicitly by using homotopy analysis method (HAM). The resulting solutions are investigated for heat transfer effects on velocity and temperature profiles.

## 1. Introduction

The flow domain, described by thin film flow, in one of the dimensions is much smaller than the flow in the other one or two dimensions. By utilizing this fact, a set of simplified modeling equations can possibly be deduced from the Navier Stokes equations. Gravity-driven laminar flow problems including the thin film flow have significant practical applications in many fields like industrial and chemical engineering, coating flows, biofluids, microfluidic engineering, and medicine. The vast majority of the investigations on different falling film flow phenomena have been studied over the years. Fulford [1] has described in detail a variety of concepts to analyze the thin film flow procedure. However, Andersson and Ytrehus [2, 3] studied the diffusion from a vertical wall into an accelerating falling liquid film and gave the Falkner-Skan solutions for gravity-driven film flows. On the other hand, Sparrow et al. [4] described the combined forced and free convection in boundary layer flow about the nonisothermal body subjected to a uniform free stream velocity, and also he gave the criteria for cataloging flows as purely forced, purely free, and mixed. A different approach was adopted by Andersson and Irgens [5, 6], namely, to divide the accelerating film flow into a developing viscous boundary layer and an external inviscid free stream. They furthermore demonstrated that a similarity transformation exists, such that the boundary layer momentum equation for power-law fluids is exactly transformed into a Falkner-Skan type ordinary differential equation. Andersson et al. [7] also investigated the effects of high Prandtl number and temperature differences in the laminar film flow with combined and natural convection. He also concluded that for the vast majority of the parameter combinations the resulting velocity profiles *f*′(*η*) increased monotonically from zero at the surface to unity in the free stream.

Our motivation to do the present work is to investigate the heat transfer due to the gravity-driven laminar film flow over the convectively heated surface. Further, both aiding and opposing buoyancy are considered to see its effect on the film flow and heat transfer. The homotopy analysis method (HAM; see [8–13]) is implied to solve the considered problem, and explicit solutions with high precision are also obtained. To our knowledge, this is the first time to explore the explicit solutions for this particular gravity-driven film flow problem with convective boundary condition. Moreover, the squared residual has been calculated which shows the correctness of our obtained explicit solutions.

## 2. Mathematical Formulation and Analysis

Consider the two-dimensional laminar film flow of Newtonian fluid along a vertical surface. That vertical surface is heated or cooled from below by convection from a fluid of temperature *T*_*f*_, while *T*_*∞*_ is the temperature outside the boundary layers. To describe this laminar film flow we must add a gravity term (body force term) to the momentum equation, and then using the boundary layer, assumptions will give rise to the following partial differential equation:

(1) $$\begin{array}{c} \frac{\partial u}{\partial x}+\frac{\partial \upsilon }{\partial y}=0, \end{array}$$

(2) $$\begin{array}{c} \rho_{0}\left[u\frac{\partial u}{\partial x}+\upsilon \frac{\partial u}{\partial y}\right]=\rho g+\mu \frac{\partial^{2}u}{\partial y^{2}}, \end{array}$$

(3) $$\begin{array}{c} \rho_{0}c_{p}\left[u\frac{\partial T}{\partial x}+\upsilon \frac{\partial T}{\partial y}\right]=k\frac{\partial^{2}T}{\partial y^{2}}, \end{array}$$

subject to boundary conditions

(4) $$\begin{array}{c} u=0,\;\;\upsilon =0,\\ -k\frac{\partial T}{\partial y}=h_{f}\left(T_{f}-T\right),\;\text{at}\;y=0,\\ u\to U\left(x\right),\;T\to T_{\infty },\;\text{as}\;y\to \infty . \end{array}$$

Here *u* is vertical velocity component along vertical *x*-axis and *υ* is horizontal velocity component along horizontal *y*-axis. Whereas *c*_*p*_ is specific heat at constant pressure, *ρ* is the fluid density, *μ* is dynamic viscosity, *k* is thermal conductivity, *g* is the gravitational constant, *h*_*f*_(*x*) is the heat transfer coefficient due to *T*_*f*_, and *U*(*x*) is free stream velocity. According to the classical boundary-layer approximations, it is assumed that stream-wise diffusion of momentum and longitudinal heat conduction is negligible in (2) and (3). Also all the properties of fluid are kept constant and the internal dissipation of energy is neglected except the density, (*ρ*(*T*)). For this purpose the Boussinesq approximation is used on the buoyancy term in the momentum equation to represent the density variation as

(5) $$\begin{array}{c} \rho =\rho_{0}\left[1-\alpha \left(T-T_{0}\right)\right], \end{array}$$

where *ρ*_0_ is the density at the arbitrary reference temperature *T*_0_ and *α* is the coefficient of thermal expansion. Since the frictionless flow between the viscous boundary layer and the free streamline bordering the constant-pressure atmosphere is considered to be irrotational with downward velocity, *U*(*x*) is quasi-one-dimensional. Assuming the infinite film thickness at the entrance *x* = 0, the simple free stream solution

(6) $$\begin{array}{c} U\left(x\right)=\sqrt{2gx} \end{array}$$

is readily derived by integrating the one-dimensional version of (2), given as

(7) $$\begin{array}{c} U\frac{dU}{dx}=g. \end{array}$$

We now introduce the following similarity transformations:

(8) $$\begin{array}{c} \psi \left(\eta \right)=f\left(\eta \right)\cdot {\left(\frac{4U\nu x}{3}\right)}^{1/2},\;\;\eta =y\cdot {\left(\frac{3U}{4\nu x}\right)}^{1/2},\\ \theta \left(\eta \right)=\frac{T-T_{\infty }}{T_{f}-T_{\infty }}, \end{array}$$

where *ν* is kinematic viscosity and *ψ* is the stream function defined as

(9) $$\begin{array}{c} u=\frac{\partial \psi }{\partial y},\;\;\upsilon =-\frac{\partial \psi }{\partial x}. \end{array}$$

The following set of equations is formed by substituting the similarity transformations from (8) to (1)–(3) and thus can be rewritten as

(10) $$\begin{array}{c} f^{\prime \prime \prime }\left(\eta \right)+f\left(\eta \right)f^{\prime \prime }\left(\eta \right)+\frac{2}{3}\left(1-f^{\prime }{\left(\eta \right)}^{2}\right)-\frac{4}{3}\lambda \theta =0,\\ \theta^{\prime \prime }\left(\eta \right)+\text{Pr}f\left(\eta \right)\theta^{\prime }\left(\eta \right)=0 \end{array}$$

with the associated boundary conditions given as

(11) $$\begin{array}{c} f\left(0\right)=0,\;\;\;f^{\prime }\left(0\right)=0,\;\;f^{\prime }\left(\infty \right)=1,\\ \theta \left(\infty \right)=0,\;\;\theta^{\prime }\left(0\right)=-\gamma \left(1-\theta \left(0\right)\right), \end{array}$$

where Pr is the Prandtl number, *γ* is reduced heat transfer parameter, and *λ* is the dimensionless temperature difference defined as *λ* ≡ Gr_*x*_/*Re*_*x*_^2^ = *α*(*T*_*f*_ − *T*_*∞*_)/2. Since the dimensionless parameters related to the measure of convection velocity are defined as the local Grashof number, Gr_*x*_ = *αg*(*T*_*f*_ − *T*_0_)*x* ^3^/*ν* ^2^, and local Reynolds number, *Re*_*x*_ = *Ux*/*ν*.

## 3. Explicit Solutions by Homotopy Analysis Method

The homotopy analysis method has been employed here to give the explicit solutions of the nonlinear coupled differential equation. As it is shown by ((A.11)) (see the Appendix), the solution series for *f*(*η*) and *θ*(*η*) can be expressed as

(12) $$\begin{array}{c} f\left(\eta \right)=f_{0}\left(\eta \right)+\sum_{m=1}^{+\infty }f_{m}\left(\eta \right),\\ \theta \left(\eta \right)=\theta_{0}\left(\eta \right)+\sum_{m=1}^{+\infty }\theta_{m}\left(\eta \right), \end{array}$$

where *f*_*m*_(*η*) and *θ*_*m*_(*η*) are higher order deformation derivatives. Now we can solve the first several higher order deformation equations ((A.12))–((A.14)) to obtain *f*_*m*_(*η*) and *θ*_*m*_(*η*), which is defined as

(13) $$\begin{array}{c} f_{m}\left(\eta \right)=A_{0,0}^{m}+\sum_{j=1}^{2m+2}\;\sum_{i=0}^{2m+2}\sigma_{i,j}^{m}\;A_{i,j}^{m}\;\eta^{i}\;\exp^{-j\beta \eta },\\ \theta_{m}\left(\eta \right)=\sum_{j=1}^{2m+2}\;‍\sum_{i=0}^{2m+2}\sigma_{i,j}^{m}\;B_{i,j}^{m}\;\eta^{i}\;\exp^{-j\beta \eta }, \end{array}$$

where *β* is the given positive constant and *σ*_*i*,*j*_^*m*^ is the coefficient defined as

(14) $$\begin{array}{c} \sigma_{i,j}^{m}=\{\begin{array}{cc} 1, & 0\leq i\leq 2m,\;\;1\leq j\leq 2m+2-\chi_{i}-2\left\lfloor \frac{i+1}{2}\right\rfloor ,\\ 0, & \text{otherwise}. \end{array} \end{array}$$

Here *χ*_*i*_ is given by ((A.17)) and ⌊·⌋ represents the integer floor function. Substituting the above expression into ((A.12))–((A.14)), we have the recursive coefficients *A*_*i*,*j*_^*m*^ and *B*_*i*,*j*_^*m*^ for *m* ≥ 1 determined as

(15) $$\begin{array}{c} A_{i,j}^{m}=\chi_{m}\sigma_{i,j}^{m-1}A_{i,j}^{m-1}+\sum_{q=i}^{2m+2}\Gamma_{q,j}^{m}\;\mu_{j,i}^{q},\\ B_{i,j}^{m}=\chi_{m}\delta_{i,j}^{m-1}B_{i,j}^{m-1}+\sum_{q=i}^{2m+2}\Psi_{q,j}^{m}\;\delta_{j,i}^{q}, \end{array}$$

for 2 ≤ *j* ≤ 2*m* + 2 and 0 ≤ *i* ≤ 2*m*,

(16) Ai,1m=χmσi,1m−1Ai,1m−1+∑q=max⁡⁡[0,i−1]2mΔq,1m μ1,iq,Bi,1m=χmδi,1m−1Bi,1m−1+∑q=max⁡⁡[0,i−1]2mΩq,1m δ1,iq,A0,0m=χmA0,0m−1+∑j=22m+2 ‍∑q=12m+2Γq,jm[(j−1)μj,0q−1βμj,1q]−∑q=02m1βΔq,1m μ1,1q+∑j=22m+2(j−1)Γ0,jm μj,00,A0,1m=χmσ0,1m−1A0,1m−1+∑j=22m+2 ‍∑q=12m+21βΓq,jm μj,1q+∑q=02m1βΔq,1m μ1,1q−∑j=22m+2jΓq,jm μj,0q,B0,1m=χmσ0,1m−1B0,1m−1−∑j=22m+2 ‍∑q=02mΨq,jm δj,0q,A0,jm=0,

where

(17) δn,jq={q!j!1(2β)q−j+2,0≤j≤q,  n=1,12β(q+1),j=q+1,  n=1,q!j!12β(1[(n+1)β]q−j+1      −1[(n−1)β]q−j+1),0≤j≤q,  n>1,0,j=q+1,  n>1,μn,jq={∑i=jq+1δn,jqβi−j+1,0≤j≤q+1,  n=1,∑i=jqδn,jq(nβ)i−j+1,0≤j≤q,  n>1,0,j=q+1,  n>1.

Other coefficients involved in the above recursive formulae are given as

(18) $$\begin{array}{c} \Gamma_{i,j}^{m}=\hbar_{f}\left({\tilde{A}}_{i,j}^{m}+{\tilde{B}}_{i,j}^{m}+{\tilde{E}}_{i,j}^{m}+J_{i,j}^{m-1}-\frac{4}{3}\lambda \sigma_{i,j}^{m-1}B_{i,j}^{m-1}\right),\\ \Delta_{i,1}^{m}=\hbar_{f}\left(J_{i,1}^{m-1}-\frac{4}{3}\lambda \sigma_{i,1}^{m-1}B_{i,1}^{m-1}+{\tilde{B}}_{i,1}^{m}\right),\\ \Psi_{i,j}^{m}=\hbar_{\theta }\left({\tilde{H}}_{i,j}^{m-1}+\text{Pr}\;{\tilde{F}}_{i,j}^{m}+\text{Pr}\;{\tilde{K}}_{i,j}^{m}\right),\\ \Omega_{i,1}^{m}=\hbar_{\theta }\left(H_{i,1}^{m-1}+\text{Pr}\;{\tilde{K}}_{i,1}^{m}\right), \end{array}$$

where *ℏ*_*f*_ and *ℏ*_*θ*_ are the convergence control parameters, and

(19) Di,jm=σi+1,jmAi+1,jm(i+1)−(jβ)σi,jmAi,jm,Ei,jm=σi+1,jmBi+1,jm(i+1)−(jβ)σi,jmBi,jm,Gi,jm=(i+2)(i+1)σi+2,jmAi+2,jm−(2jβ)(i+1)σi+1,jmAi+1,jm+(jβ)2σi,jmAi,jm,Hi,jm=(i+2)(i+1)σi+2,jmBi+2,jm−(2jβ)(i+1)σi+1,jmBi+1,jm+(jβ)2σi,jmBi,jm,Ji,jm=(i+3)(i+2)(i+1)σi+3,jmAi+3,jm−(3jβ)(i+2)(i+1)σi+2,jmAi+2,jm+3(i+1)(jβ)2σi+1,jmAi+1,jm−(jβ)3σi,jmAi,jm,Ki,jm=(i+3)(i+2)(i+1)σi+3,jmBi+3,jm−(3jβ)(i+2)(i+1)σi+2,jmBi+2,jm+3(i+1)(jβ)2σi+1,jmBi+1,jm−(jβ)3σi,jmBi,jm.

Also,

(20) $$\begin{array}{c} {\tilde{A}}_{i,j}^{m}=\sum_{n=0}^{m-1}\;‍\sum_{s=\max\left[1,j-2n-2\right]}^{\min\left[2m-2n,j-1\right]}\;‍\sum_{r=\max\left[1,i-2n-2\right]}^{\min\left[2m-2n,i\right]}\sigma_{r,s}^{m-1-n}A_{r,s}^{m-1-n}G_{i-r,j-s}^{n},\\ {\tilde{B}}_{i,j}^{m}=\sum_{n=\max\left[j-1,i-1\right]}^{m-1}A_{0,0}^{m-1-n}G_{i,j}^{n},\\ {\tilde{E}}_{i,j}^{m}=\sum_{n=0}^{m-1}\;‍\sum_{s=\max\left[1,j-2n-2\right]}^{\min\left[2m-2n,j-1\right]}\;‍\sum_{r=\max\left[1,i-2n-2\right]}^{\min\left[2m-2n,i\right]}D_{r,s}^{m-1-n}D_{i-r,j-s}^{n},\\ {\tilde{F}}_{i,j}^{m}=\sum_{n=0}^{m-1}\;‍\sum_{s=\max\left[1,j-2n-2\right]}^{\min\left[2m-2n,j-1\right]}\;‍\sum_{r=\max\left[1,i-2n-2\right]}^{\min\left[2m-2n,i\right]}\sigma_{r,s}^{m-1-n}A_{r,s}^{m-1-n}E_{i-r,j-s}^{n},\\ {\tilde{K}}_{i,j}^{m}=\sum_{n=\max[j-1,i-1]}^{m-1}A_{0,0}^{m-1-n}E_{i,j}^{n}. \end{array}$$

Using all the above recursive formulae and setting *A*_0,0_^0^ = 1, *A*_0,1_^0^ = −1/*β*, *A*_0,2_^0^ = 1/*β*, *B*_0,1_^0^ = 1, and *B*_0,2_^0^ = −*β*/(*γ* + 2*β*), we can calculate all the coefficients, and thus purely explicit analytical solution for *f*(*η*) and *θ*(*η*) can be obtained for *m* = 1,2, 3,….

## 4. Results and Discussions

It is well known that convergence of the HAM series, given by ((A.1)), not only depends upon the proper choice of initial guess or linear operator but it also relies on the proper value of convergence-control parameter. Basically we can select the appropriate values of auxiliary parameters by two methods: first by plotting the *ℏ*-curves [10] and then choosing its value in the corresponding valid regions of *ℏ* and second by determining the minimum of the squared residual [9] of the governing equation which gives the appropriate value of convergence-control parameter. However, one can also accelerate the convergence of homotopy series by using the homotopy-Pade technique or homotopy-iterative approach [9] for the problems with strong nonlinearity.

In order to check the validity of our gained explicit solutions, we apply the second approach by defining the discrete squared residual for *f*(*η*) and *θ*(*η*) as

(21) $$\begin{array}{c} \text{Er}{\text{r}}_{f}^{m}=\frac{1}{k+1}\sum_{j=0}^{k}{\left[N_{f}\left(\sum_{n=0}^{m}f_{n}\right)\right]}^{2}d\eta \\ \text{Er}{\text{r}}_{\theta }^{m}=\frac{1}{k+1}\sum_{j=0}^{k}{\left[N_{f}\left(\sum_{n=0}^{m}\theta_{n}\right)\right]}^{2}d\eta , \end{array}$$

where *k* is an integer. *N*_*f*_ and *N*_*θ*_ denote the nonlinear operators given by ((A.7)). Here we use *k* = 40 to gain the computational error. It is pertinent to mention here that using discrete squared residual reduces the CPU time, suggested in [9, 14] rather than finding exact square residual of the governing equations. Especially, it is convenient to use it when there is more than one unknown auxiliary parameter involved.

Further, one can improve the convergence of obtained results by introducing more convergence-control parameters in the frame of HAM. In particular, we have used another convergence-control parameter induced in the linear operator and initial guess denoted by *β*. The other two convergence-control parameters are *ℏ*_*f*_ and *ℏ*_*θ*_. The optimal values for the *β*, *ℏ*_*f*_, and *ℏ*_*θ*_ are determined by evaluating the minimum error of (21). In our case the value of convergence-control parameters is taken to be *ℏ*_*f*_ = −1/2 and *ℏ*_*θ*_ = −1/2. Also, here the selected value for third parameter is *β* = 5, which has greatly accelerated the convergence of our series solution by decreasing the average square residual. The reduction in the error becomes quite slow as compared to our present case if we change the value of *β*, and even in some cases the results are not convergent. So, we can always reduce the error and can control the convergence of HAM analytical approximations by choosing the proper values of convergence-control parameters.

Obviously, for given order of approximation *m*, if the value of Err_*f*_^*m*^ and Err_*θ*_^*m*^ is smaller, the better is the approximation. To confirm this, we calculated the error for *f*(*η*) given in Tables 1 and 2 for different values of *γ* and *λ*. Similarly the error for *θ*(*η*) is shown in Tables 3 and 4. It is also noted that Err_*f*_^*m*^ and Err_*θ*_^*m*^ decrease more quickly in case of favorable buoyancy, which approached 1.6 × 10^−5^ and 2.1 × 10^−5^ at 40th-order approximation for several values of *γ*. The reduction in the error for each case with the increasing number of iterations shows the reliability of our analytic results. Also the accuracy of these results can be improved far more by increasing the order of approximation.

**Figure 1:**
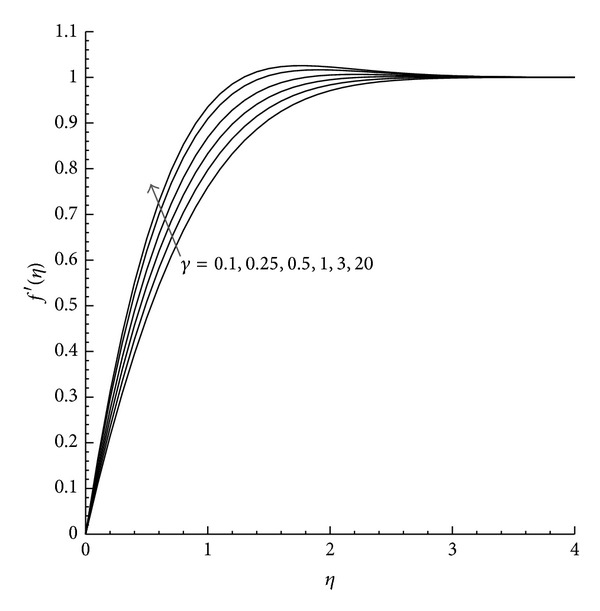
Velocity profile *f*′(*η*) for different values of *γ* where *λ* = −1 and Pr = 1.

**Figure 2:**
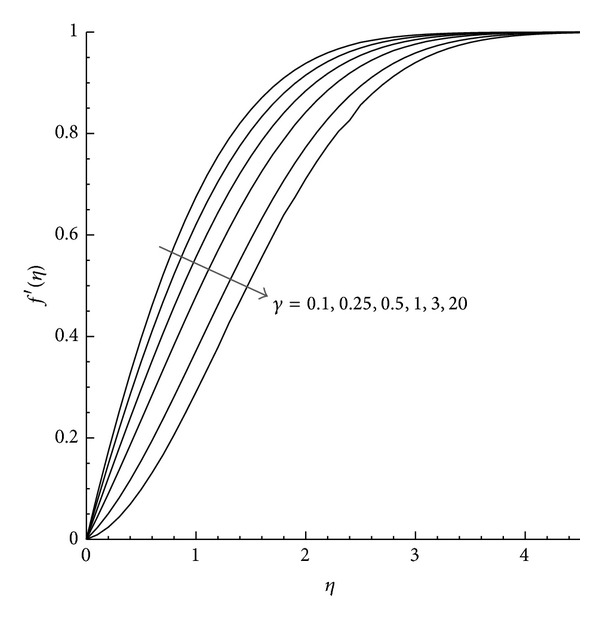
Velocity profile *f*′(*η*) for different values of *γ* where *λ* = 1 and Pr = 1.

**Figure 3:**
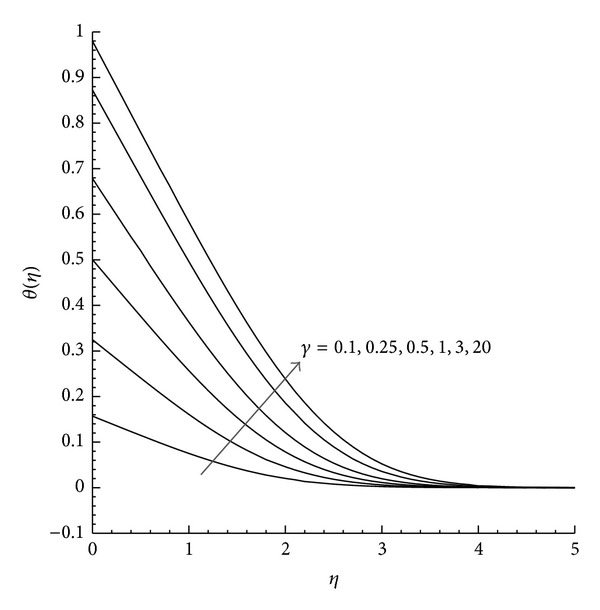
Temperature profile *θ*(*η*) for different values of *γ* where *λ* = 1 and Pr = 1.

**Figure 4:**
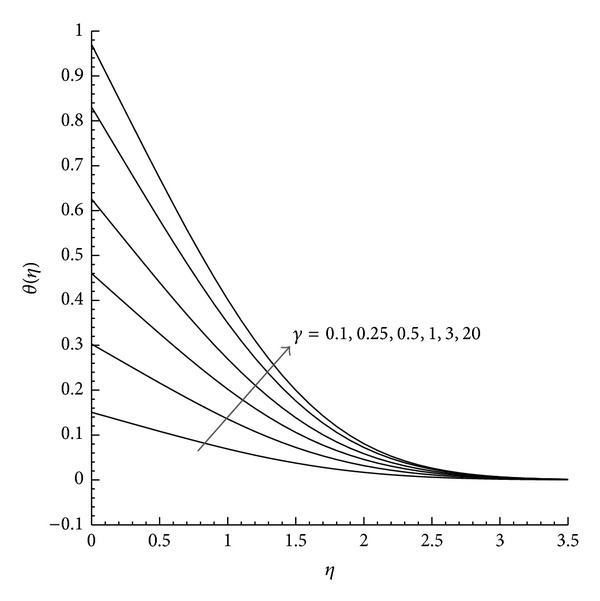
Temperature profile *θ*(*η*) for different values of *γ* where *λ* = −1 and Pr = 1.

Moreover, the velocity and temperature profiles are plotted for favorable (*λ* = −1) and unfavorable (*λ* = +1) buoyancy with the variation of *γ*.  Figure 1 shows that in the case of favorable buoyancy, as the value of *γ* increases the velocity component increases with the increase in *η* but reduces by unfavorable buoyancy as presented in Figure 2. Further the temperature profile observed from Figures 3 and 4 describes the effect of *γ* with the temperature differences (|*λ* | = 1). From these figures we can examine that as *η* increases the temperature decreases, while the increase in the reduced heat transfer coefficient *γ* increases *θ*(*η*) for both aiding and opposing buoyancy.

## 5. Conclusions

The explicit solutions are obtained in this paper using HAM for the gravity-driven film flow over a vertical impermeable sheet with convective boundary condition. By choosing the appropriate value for the convergence-control parameters we obtain the error for *f*(*η*) and *θ*(*η*). The decrease in the error with the increase in the number of approximations depicts the validity of our analytic solutions. According to the authors' view about such kind of analytical solutions, they have never been presented in the literature before. Finally, this is due to homotopy analysis method that we are able to give quite accurate explicit solutions by choosing the proper values of convergence-control parameters, base functions, initial approximations, and linear operators.

## Figures and Tables

**Table 1:** Average squared residual errors for Err_*f*_^*m*^ with *ℏ*_*f*_ = −1/2, ℏ_θ_ = −1/2, and β = 5 in the case of Pr = 1 and λ = 1.

*k*th order	γ = 1	γ = 5	γ = 10	γ = 15	γ = 20
5	2.2555 × 10^−1^	2.48030 × 10^−1^	2.56497 × 10^−1^	2.60104 × 10^−1^	2.62083 × 10^−1^
10	6.91281 × 10^−2^	8.82655 × 10^−2^	9.43782 × 10^−2^	9.66925 × 10^−2^	9.78824 × 10^−2^
20	5.043001 × 10^−3^	1.00880 × 10^−2^	1.12832 × 10^−2^	1.17021 × 10^−2^	1.19141 × 10^−2^
30	8.19099 × 10^−4^	2.58524 × 10^−3^	3.04192 × 10^−3^	3.21354 × 10^−3^	3.30340 × 10^−3^
40	1.69486 × 10^−4^	9.09989 × 10^−4^	1.14358 × 10^−3^	1.23538 × 10^−3^	1.28426 × 10^−3^

**Table 2:** Average squared residual errors for Err_*f*_^*m*^ with ℏ_*f*_ = −1/2, *ℏ*_θ_ = −1/2, and β = 5 in the case of Pr = 1 and λ = −1.

*k*th order	γ = 1	γ = 5	γ = 10	γ = 15	γ = 20
5	1.11846 × 10^−1^	9.74131 × 10^−2^	9.26109 × 10^−2^	9.06737 × 10^−2^	8.96424 × 10^−2^
10	1.09109 × 10^−2^	6.67928 × 10^−3^	5.51535 × 10^−3^	5.09535 × 10^−3^	4.88484 × 10^−3^
20	3.02345 × 10^−4^	6.20106 × 10^−4^	6.00372 × 10^−4^	5.82802 × 10^−4^	5.71778 × 10^−4^
30	8.14679 × 10^−5^	1.45809 × 10^−4^	1.45809 × 10^−4^	1.35611 × 10^−4^	1.32648 × 10^−4^
40	9.69675 × 10^−6^	1.70644 × 10^−5^	1.70644 × 10^−5^	1.58480 × 10^−5^	1.54868 × 10^−5^

**Table 3:** Average squared residual errors for Err_*θ*_^*m*^ with ℏ_*f*_ = −1/2, ℏ_θ_ = −1/2, and β = 5 in the case of Pr = 1 and λ = 1.

*k*th order	γ = 1	γ = 5	γ = 10	γ = 15	γ = 20
5	5.31587 × 10^−2^	9.32074 × 10^−2^	1.19486 × 10^−1^	1.33022 × 10^−1^	1.41151 × 10^−1^
10	1.81487 × 10^−2^	4.38441 × 10^−2^	5.34957 × 10^−2^	5.68101 × 10^−2^	5.83709 × 10^−2^
20	2.81659 × 10^−3^	7.36339 × 10^−3^	8.33899 × 10^−3^	8.64952 × 10^−3^	8.80023 × 10^−3^
30	4.96569 × 10^−4^	1.62656 × 10^−3^	1.91199 × 10^−3^	2.01813 × 10^−3^	2.07356 × 10^−3^
40	9.44952 × 10^−5^	4.78105 × 10^−4^	6.01700 × 10^−4^	6.50358 × 10^−4^	6.76279 × 10^−4^

**Table 4:** Average squared residual errors for Err_*θ*_^*m*^ with ℏ_*f*_ = −1/2, ℏ_θ_ = −1/2, and β = 5 in the case of Pr = 1 and λ = −1.

*k*th order	γ = 1	γ = 5	γ = 10	γ = 15	γ = 20
5	5.01248 × 10^−2^	8.58652 × 10^−2^	1.10077 × 10^−1^	1.22631 × 10^−1^	1.30186 × 10^−1^
10	1.37880 × 10^−2^	3.10149 × 10^−2^	3.73286 × 10^−2^	3.92840 × 10^−2^	4.01227 × 10^−3^
20	1.10969 × 10^−3^	2.08900 × 10^−3^	2.24156 × 10^−3^	2.25752 × 10^−3^	2.25533 × 10^−3^
30	1.00475 × 10^−4^	1.92774 × 10^−4^	1.86875 × 10^−4^	1.80412 × 10^−4^	1.76338 × 10^−4^
40	8.43581 × 10^−6^	2.22668 × 10^−5^	2.18639 × 10^−5^	2.11068 × 10^−5^	2.06069 × 10^−5^

## Appendix

According to the boundary conditions (11) we can express the solution of *f*(*η*) and *θ*(*η*) as follows:

(A.1) $$\begin{array}{c} f\left(\eta \right)=A_{0,0}+\sum_{n=1}^{\infty }\;‍\sum_{m=0}^{\infty }A_{m,n}\;\eta^{m}\;\exp^{-n\beta \eta },\\ \theta \left(\eta \right)=\sum_{n=1}^{\infty }\;‍\sum_{m=0}^{\infty }B_{m,n}\;\eta^{m}\;\exp^{-n\beta \eta }, \end{array}$$

where *A*_*m*,*n*_ and *B*_*m*,*n*_ are the coefficients. Due to the solution expressions given by (A.1) and boundary conditions (11), we can choose

(A.2) $$\begin{array}{c} f_{0}\left(\eta \right)=\eta +\frac{\exp^{-2\beta \eta }-\exp^{-\beta \eta }}{\beta },\\ \theta_{0}\left(\eta \right)=\exp^{-\beta \eta }+\frac{\beta }{2\beta +\gamma }\exp^{-2\beta \eta } \end{array}$$

as initial guess for *f*(*η*) and *θ*(*η*). The auxiliary linear operator corresponding to (10) is given as

(A.3) $$\begin{array}{c} L_{f}=\frac{\partial^{3}}{\partial \eta^{3}}+\beta \frac{\partial^{2}}{\partial \eta^{2}},\;\;L_{\theta }=\frac{\partial^{2}}{\partial \eta^{2}}+\beta \frac{\partial }{\partial \eta } \end{array}$$

with the property

(A.4) $$\begin{array}{c} L_{f}\left[C_{1}\exp^{-\beta \eta }+C_{2}+C_{3}\eta \right]=0,\\ L_{\theta }\left[C_{1}\exp^{-\beta \eta }+C_{2}\right]=0, \end{array}$$

where *C*_1_, *C*_2_, and *C*_3_ are integral constants. Let *ℏ*_*f*_ and *ℏ*_*θ*_ denote the convergence control parameters for *f*(*η*) and *θ*(*η*), respectively. Now we will make the zeroth-order deformation equation

(A.5) $$\begin{array}{c} \left(1-q\right)L_{f}\left[F\left(\eta ;q\right)-f_{0}\left(\eta ;q\right)\right]=q\hbar_{f}N_{f}\left[F\left(\eta ;q\right),\Theta \left(\eta ;q\right)\right],\\ \left(1-q\right)L_{\theta }\left[\Theta \left(\eta ;q\right)-\theta_{0}\left(\eta ;q\right)\right]=q\hbar_{\theta }N_{\theta }\left[F\left(\eta ;q\right),\Theta \left(\eta ;q\right)\right] \end{array}$$

subject to the boundary conditions

(A.6) $$\begin{array}{c} F\left(0;q\right)={\frac{\partial F\left(\eta ;q\right)}{\partial \eta }|}_{\eta =0}=0,\;\;{\frac{\partial F\left(\eta ;q\right)}{\partial \eta }|}_{\eta =\infty }=1,\\ \Theta \left(\infty ,q\right)=0,\;\;{\frac{\partial \Theta \left(\eta ;q\right)}{\partial \eta }|}_{\eta =0}=-\gamma \left(1-\Theta \left(0\right)\right), \end{array}$$

where the nonlinear operators *N*_*f*_ and *N*_*θ*_ are defined by using (10) as

(A.7) $$\begin{array}{c} N_{f}=\frac{\partial^{3}F}{\partial \eta^{3}}+F\frac{\partial^{2}F}{\partial \eta^{2}}+\frac{2}{3}\left(1-{\left(\frac{\partial F}{\partial \eta }\right)}^{2}\right)-\frac{4}{3}\lambda \Theta ,\\ N_{\theta }=\frac{\partial^{2}\Theta }{\partial \eta^{2}}+\text{Pr}\cdot F\frac{\partial \Theta }{\partial \eta }, \end{array}$$

where *q* is an embedding parameter and *F*(*η*; *q*) and Θ(*η*; *q*) are real functions of *η* and *q*. Obviously, when *q* = 0 and *q* = 1, it is clear from (10) and the above zeroth-order deformation equations given by (A.5) that

(A.8) $$\begin{array}{c} F\left(\eta ;0\right)=f_{0}\left(\eta \right),\;\;\Theta \left(\eta ;0\right)=\theta_{0}\left(\eta \right),\\ F\left(\eta ;1\right)=f\left(\eta \right),\;\;\Theta \left(\eta ;1\right)=\theta \left(\eta \right). \end{array}$$

So, as *q* increases from 0 to 1, *F*(*θ*; *q*) and Θ(*η*; *q*) vary from the initial guesses *f*_0_(*η*) and *θ*_0_(*η*) to the corresponding exact solutions *f*(*η*) and *θ*(*η*).

Expanding *F*(*η*; *q*) and Θ(*η*; *q*) in Taylor's series at *q* = 0, we have

(A.9) $$\begin{array}{c} F\left(\eta ;q\right)=F\left(\eta ;0\right)+\sum_{m=1}^{+\infty }f_{m}\left(\eta \right)q^{m},\\ \Theta \left(\eta ;q\right)=\Theta \left(\eta ;0\right)+\sum_{m=1}^{+\infty }\theta_{m}\left(\eta \right)q^{m}, \end{array}$$

where

(A.10) $$\begin{array}{c} f_{m}\left(\eta \right)=\frac{1}{m!}{\frac{\partial^{m}F\left(\eta ;q\right)}{\partial q^{m}}|}_{q=0},\\ \theta_{m}\left(\eta \right)=\frac{1}{m!}{\frac{\partial^{m}\Theta \left(\eta ;q\right)}{\partial q^{m}}|}_{q=0}. \end{array}$$

Assume that all the HAM parameters are so properly chosen that the series defined by (A.9) converges at *q* = 1. Then using (A.8) we get the solution series as

(A.11) $$\begin{array}{c} f\left(\eta \right)=f_{0}\left(\eta \right)+\sum_{m=1}^{+\infty }f_{m}\left(\eta \right),\\ \theta \left(\eta \right)=\theta_{0}\left(\eta \right)+\sum_{m=1}^{+\infty }\theta_{m}\left(\eta \right). \end{array}$$

Now differentiating the zeroth-order deformation equations (A.5), *m*-times with respect to *q*, then setting *q* = 0, and finally dividing with *m*!, we obtained the *m*th-order deformation equations for *f*_*m*_(*η*) and *θ*_*m*_(*η*):

(A.12) $$\begin{array}{c} L_{f}\left[f_{m}\left(\eta \right)-\chi_{m}f_{m-1}\left(\eta \right)\right]=\hbar_{f}R_{f}^{m}\left(\eta \right), \end{array}$$

(A.13) $$\begin{array}{c} L_{\theta }\left[\theta_{m}\left(\eta \right)-\chi_{m}\theta_{m-1}\left(\eta \right)\right]=\hbar_{\theta }R_{\theta }^{m}\left(\eta \right) \end{array}$$

subject to boundary conditions

(A.14) $$\begin{array}{c} f_{m}\left(0\right)=0,\;\;f_{m}^{\prime }\left(0\right)=0,\;\;f_{m}^{\prime }\left(\infty \right)=0,\\ \theta_{m}\left(\infty \right)=0,\;\;\theta_{m}^{\prime }\left(0\right)=\gamma \theta_{m}\left(0\right), \end{array}$$

where

(A.15) Rfm(η)=fm−1′′′(η)+∑n=0m−1fn(η)fm−1−n′′(η) −23∑n=0m−1fn′(η)fm−1−n′(η) −43λθm−1(η)+23(1−χm),

(A.16) $$\begin{array}{c} R_{\theta }^{m}\left(\eta \right)=\theta_{m-1}^{\prime \prime }\left(\eta \right)+\text{Pr}\sum_{n=0}^{m-1}f_{n}\left(\eta \right)\theta_{m-1-n}^{\prime }\left(\eta \right), \end{array}$$

(A.17) $$\begin{array}{c} \chi_{m}=\{\begin{array}{cc} 0, & m\leq 1,\\ 1, & m>1. \end{array} \end{array}$$

## References

[1] Fulford GD, Drew TB, Hoopes JW, Vermeulen T (1964). The flow of liquids in thin films. *Advances in Chemical Engineering*.

[2] Andersson HI (1987). Diffusion from a vertical wall into an accelerating falling liquid film. *International Journal of Heat and Mass Transfer*.

[3] Andersson HI, Ytrehus T (1985). Falkner-Skan solution for gravity-driven film flow. *Journal of Applied Mechanics*.

[4] Sparrow EM, Eichhorn R, Gregg JL (1959). Combined forced and free convection in a boundary layer flow. *Physics of Fluids*.

[5] Andersson HI, Irgens F (1988). Gravity-driven laminar film flow of power-law fluids along vertical walls. *Journal of Non-Newtonian Fluid Mechanics*.

[6] Andersson HI, Irgens F, Cheremisinoff NP (1990). Film flow of power-law fluids. *Encyclopedia of Fluid Mechanics*.

[7] Andersson HI, Pettersson BA, Dandapat BS (1994). Combined forced and natural convection in laminar film flow. *Wärme- und Stoffübertragung*.

[8] Liao S (1999). An explicit, totally analytic approximate solution for Blasius’ viscous flow problems. *International Journal of Non-Linear Mechanics*.

[9] Liao SJ (2012). *Homotopy Analysis Method in Nonlinear Differential Equations*.

[10] Liao SJ (2003). *Beyond Perturbation: Introduction to the Homotopy Analysis Method*.

[11] Liao S, Pop I (2004). Explicit analytic solution for similarity boundary layer equations. *International Journal of Heat and Mass Transfer*.

[12] Xu H (2004). An explicit analytic solution for free convection about a vertical flat plate embedded in a porous medium by means of homotopy analysis method. *Applied Mathematics and Computation*.

[13] Wang C, Zhu JM, Liao SJ, Pop I (2003). On the explicit analytic solution of Cheng-Chang equation. *International Journal of Heat and Mass Transfer*.

[14] Liao S (2010). An optimal homotopy-analysis approach for strongly nonlinear differential equations. *Communications in Nonlinear Science and Numerical Simulation*.

